# Patterns of initiation of second generation antipsychotics for bipolar disorder: a month-by-month analysis of provider behavior

**DOI:** 10.1186/s12888-014-0339-z

**Published:** 2014-11-30

**Authors:** Christopher J Miller, Mingfei Li, Robert B Penfold, Austin F Lee, Eric G Smith, David N Osser, Laura Bajor, Mark S Bauer

**Affiliations:** The Center for Healthcare Organization and Implementation Research, VA Boston Healthcare System and Edith Nourse Rogers Memorial VA Medical Center, Bedford, MA USA; Department of Psychiatry, Harvard Medical School, Boston, MA USA; Department of Mathematical Sciences, Bentley University, Waltham, MA USA; Group Health Research Institute, Seattle, WA USA; Department of Health Services Research, University of Washington, Seattle, WA USA; Research Center for Medical Statistics and Actuarial Science, Xi’an University of Finance and Economics, Xi’an, China; Massachusetts General Hospital, Boston, MA USA; Department of Psychiatry, University of Massachusetts Medical School, Worcester, MA USA; VA Boston Healthcare System, Brockton, MA USA

**Keywords:** Bipolar disorder, Antipsychotics, Veterans

## Abstract

**Background:**

Several second generation antipsychotics (SGAs) received FDA approval for bipolar disorder in the 2000s. Although efficacious, they have been costly and may cause significant side effects. Little is known about the factors associated with prescribers’ decisions to initiate SGA prescriptions for this condition.

**Methods:**

We gathered administrative data from the Department of Veterans Affairs on 170,713 patients with bipolar disorder between fiscal years 2003–2010. Patients without a prior history of taking SGAs were considered eligible for SGA initiation during the study (n =126,556). Generalized estimating equations identified demographic, clinical, and comorbidity variables associated with initiation of an SGA prescription on a month-by-month basis.

**Results:**

While the number of patients with bipolar disorder using SGAs nearly doubled between 2003 and 2010, analyses controlling for patient characteristics and the rise in the bipolar population revealed a 1.2% annual decline in SGA initiation during this period. Most medical comorbidities were only modestly associated with overall SGA initiation, although significant differences emerged among individual SGAs. Several markers of patient severity predicted SGA initiation, including previous hospitalizations, psychotic features, and a history of other antimanic prescriptions; these severity markers became less firmly linked to SGA initiation over time. Providers in the South were somewhat more likely to initiate SGA treatment.

**Conclusions:**

The number of veterans with bipolar disorder prescribed SGAs is rising steadily, but this increase appears primarily driven by a corresponding increase in the bipolar population. Month-by-month analyses revealed that higher illness severity predicted SGA initiation, but that this association may be weakening over time.

## Background

Bipolar disorder is associated with high morbidity, mortality, health care costs, and risk of suicide [1–3]. Beginning in the 1960’s, lithium emerged as the frontline treatment for this disorder [4], with anticonvulsants like carbamazepine [5] and valproate [6] expanding the armamentarium. More recently, several second-generation antipsychotics (SGAs) have received FDA approval and guideline endorsement for various phases of bipolar disorder including mania, depression, and mixed states [7–10], with further support from meta-analyses [11–14]. Some treatment guidelines, in fact, go further in recommending SGAs as frontline treatments for mania and bipolar depression [15,16]. The number of annual SGA prescriptions for bipolar disorder now rivals that written for lithium and valproate combined [17]. However, several SGAs have concerning cardiometabolic side effect profiles [18–21] and they have been among the most costly classes of medications in the U.S. [22].

Little research has investigated the factors affecting providers’ decisions to prescribe SGAs in this population. Patient-based studies of mixed diagnostic groups have found inconsistent results regarding impacts of patient gender [23–32], race [23–37], or age [23,25–29,31,32,35] on SGA prescribing. Fewer studies have examined clinical factors like substance use, cardiometabolic risk, or prior treatment [23–25,27–30]. Crucially, only a small subset of these studies [33,34] focused on bipolar disorder specifically. More work is needed to identify the factors that affect providers’ decisions to treat bipolar disorder with SGAs in order to facilitate personalization of treatment and system-level quality improvement interventions.

Accordingly, this study used national Department of Veterans Affairs (VA) data to identify demographic and clinical factors associated with the decision to initiate an SGA for bipolar disorder from 2003–2010, and also to determine whether SGAs have been used more broadly (i.e. for less severely ill patients) over time. While our primary analyses focused on SGAs as a group, we secondarily investigated predictors of initiation for individual SGAs. Our analyses were designed to assess patient characteristics on a month-by-month basis, approximating the data that a prescriber might have readily accessible in deciding whether or not to prescribe an SGA.

## Methods

The VA Central Institutional Review Board approved all study procedures.

### Population

We obtained administrative records from the VA Corporate Data Warehouse for fiscal years 2003–2010. All VA service users who received a diagnosis of bipolar disorder (ICD-9 code 296.xx, including bipolar type I, type II, and Not Otherwise Specified [NOS]) at one inpatient or two outpatient service contacts in a one-year period were included in the study population. Individuals with any schizophrenia spectrum diagnosis (290.0-298.9) at any point during the study period were excluded.

From this overall sample (n = 170,713), we identified on a month-by-month basis those patients “at risk” for SGA initiation, defined as (a) not having received an SGA prescription from the beginning of the study period to the month in question (i.e., excluding those with any prior SGA treatment from 2003 to that month), and (b) having a clinical encounter for their bipolar disorder in that month. To assess characteristics likely to impact clinical decision-making at a given point in time, this sample (n = 126,556 unique individuals) was then characterized on a month-by-month basis regarding demographic and clinical characteristics and whether they had initiated an SGA in that month, as defined below.

Preliminary analyses also explored the number of providers prescribing SGAs within VA. Providers “at risk” for prescribing an SGA within a given month were defined as clinicians from prescribing specialties (physician, advanced practice nurse, physician’s assistant, PharmD) who provided a bipolar disorder diagnosis for at least one clinical encounter that month.

### Definition of SGA initiation

SGA initiation, our primary outcome, was coded dichotomously each study month. Analyses focused on the five most common oral SGAs used to treat bipolar disorder: aripiprazole, olanzapine, quetiapine, risperidone, and ziprasidone. Initial analyses investigated these medications as a group; while additional analyses assessed individual agents. Newer antipsychotics (paliperidone, iloperidone, lurasidone, asenapine), approved after 2006, were not included, nor was clozapine (due to very low prevalence within VA) [38], or injectable antipsychotics (due to a lack of adequate recording in VA administrative data).

Analyses focused on each patient’s first intentional trial of an SGA during 2003–2010 [39]. *Intentional trials* were defined as receipt of (a) at least one 30-day outpatient prescription, or (b) at least three consecutive days of inpatient administration. Thirty days was the modal duration for outpatient prescriptions; sensitivity analyses reflected little variation in usage based on this duration (e.g. only 6-8% of outpatient SGA prescriptions were for <30 days). Those initiating SGAs who were hospitalized for <3 days were adequately identified by 30-day outpatient prescriptions at discharge. We included any daily dose since prescribers might start a medication at lower dosages before titrating.

For each month, patients were identified as *SGA initiators* if they (a) had not received a previous intentional SGA trial during the study period, and (b) received an intentional trial that month for any of the five study SGAs. The comparison group of *SGA non-initiators* for each month was defined as all patients who (a) had not received a previous intentional SGA trial during the study period, (b) had been seen by a prescriber for bipolar disorder that month, but who (c) did not receive a prescription for an intentional SGA trial that month. For 2003 we began analyses at month seven, thus requiring at least a six-month clean period for all patients.

### Covariates

#### Demographics

Demographic characteristics included patient age, gender, race/ethnicity, marital status, VA eligibility status (≥50% VA service-connected pension, which relieves copays for clinical services), and geographic region [40,41].

#### Clinical course variables

Bipolar type was dichotomized as bipolar type I versus bipolar type II/NOS, with bipolar I assigned if ≥10% of prior diagnoses were bipolar I; sensitivity analysis indicated that varying this cut-off from 10-50% resulted in little change in proportion of type I versus II/NOS. Psychotic features were identified for a given month if any diagnoses of mood episode with psychotic features (ICD-9 codes 296.×4) were identified over the prior year. Treatment variables included prescriptions of antidepressants and antimanic agents (lithium, anticonvulsants, first-generation antipsychotics) within the prior year. Prior hospitalization status was dichotomized as any/no prior acute mental health inpatient hospitalizations since the beginning of the study.

#### Comorbidities

Based on published literature, comorbidities judged to potentially affect provider decisions were coded as present if the patient received treatment for a given diagnosis in the 12 months prior to the month in question. These included substance use disorders, tobacco use disorder, anxiety disorder, sleep disorders, traumatic brain injury, diabetes, hyperlipidemia, cardiac dysrhythmia, and liver, kidney, or thyroid disorders.

### Establishing time-varying covariates

Some covariates (e.g. gender, race) were treated as time-invariant. Others (e.g. age, clinical course variables, comorbidities) were assessed on a month-to-month basis. This approach allowed us to characterize status for each patient for each study month. For instance, consider a hypothetical patient who was diagnosed as bipolar type I in January, 2004, that the case is not referring to an actual patient developed diabetes in January, 2005, and initiated aripiprazole in June, 2005. For any month in 2004 in which he was treated for bipolar disorder, he would qualify as an SGA non-initiator with bipolar type I. In 2005 he would remain a non-initiator, but would also carry a diabetes diagnosis. In June of 2006 he would qualify as an SGA initiator with bipolar type I and diabetes, and during subsequent months would be considered ineligible for initiator or non-initiator status.

### Data analyses

We first calculated the annual number and rate of SGA prescriptions in the bipolar population, as well as the annual number and rate of providers writing SGA prescriptions. We used preliminary time series analyses to explore the overall change in these numbers over time.

Our primary analytic tool was multivariate generalized linear modeling (GLM) with binomial distribution and logit link function to determine rates and correlates of SGA initiation on a month-by-month basis. Generalized estimating equations (GEE) were used to account for correlations from repeated measures within patients, as an individual patient could be in the dataset at several points as non-initiator before finally initiating an SGA in a later month. This allowed us to aggregate data across all 96 study months, accounting for patients appearing in multiple consecutive or non-consecutive months as described above. All variables were entered into the model simultaneously. We then added predictor-by-time interaction terms to the GLM to investigate changes over time in SGA initiation rates for those with milder versus more severe forms of bipolar disorder, specifically: (a) bipolar type I versus II/NOS, (b) psychotic features, (c) prior psychiatric hospitalization, and (d) treatment with antimanic medications.

Finally, we used multinomial univariate regression models to determine whether several comorbid conditions, characterized on a month-by-month basis, were associated with the choice of specific SGAs. The comparison drug for these analyses was aripiprazole, which is associated with relatively mild cardiometabolic or sedative effects [42]. If a patient initiated more than one SGA, only their first initiation was included in these latter analyses.

## Results

### Overall SGA use for bipolar disorder

**Figure 1:**
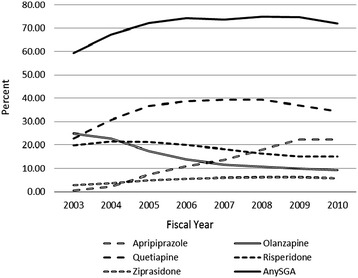
**Percent of patients with bipolar disorder treated with individual SGAs, 2003–2010.**

The number of patients with bipolar disorder prescribed SGAs almost doubled from 2003 to 2010 (31,779 in 2003; 61,697 in 2010; average annual increase of 4,226; 95% CI = 3,752-4,699; *p* < .0001), including both new initiators of these medications as well as patients continuing previous prescriptions. The number of patients treated for bipolar disorder increased more modestly (53,591 in 2003; 85,684 in 2010). Accordingly, the proportion of bipolar patients using an SGA rose from 59.3% in 2003 to a peak of 74.9% in 2008, followed by a slight decrease to 72.0% in 2010 (Figure 1; average 7.1% per year, OR = 1.071, 95% CI = 1.068-1.074, *p* < .0001. This increase was matched by a rise in the number of providers writing these prescriptions, ranging from 11,031 in 2003 to a peak of 15,965 in 2009, followed by a slight reduction to 15,609 in 2010 (average annual increase 556; 95% CI = 365-747; *p* = .0007). All told, out of our overall sample of 170,713 patients with bipolar disorder, 113,510 had at least one SGA prescription written during the study period (66%). While the number of providers writing SGA prescriptions for bipolar disorder increased, the proportion of eligible providers writing SGA prescriptions decreased slightly over the course of the study (from 90.2% in 2004 to 85.6% in 2010; annual OR = 0.927; 95% CI = 0.918-0.936; *p* < .0001).

There was heterogeneity in overall use of individual SGAs, with significant average annual increases for aripiprazole (2,910 users; 95% CI = 2,505-3,316; p < .0001), quetiapine (2,427; 95% CI = 1,571-3,284; p = .0004), and ziprasidone (534, 95% CI = 431-637; p < .0001), but a reduction for olanzapine (861 fewer annual users; 95% CI = 438-1,284; p = .0025) and no change for risperidone (138; 95% CI = −111-386; p = .2232). In terms of proportion of users, annual increases were seen for aripiprazole (OR = 1.408, 95% CI = 1.402-1.414), quetiapine (OR = 1.055, 95% CI = 1.052-1.057), and ziprasidone (OR = 1.092, 95% CI = 1.086-1.098), with decreases for olanzapine (OR = 0.832, 95% CI = 0.829-0.834) and risperidone (OR = 0.934, 95% CI = 0.931-0.936) (all *p* < 0.0001).

### SGA initiation in bipolar disorder

**Figure 2:**
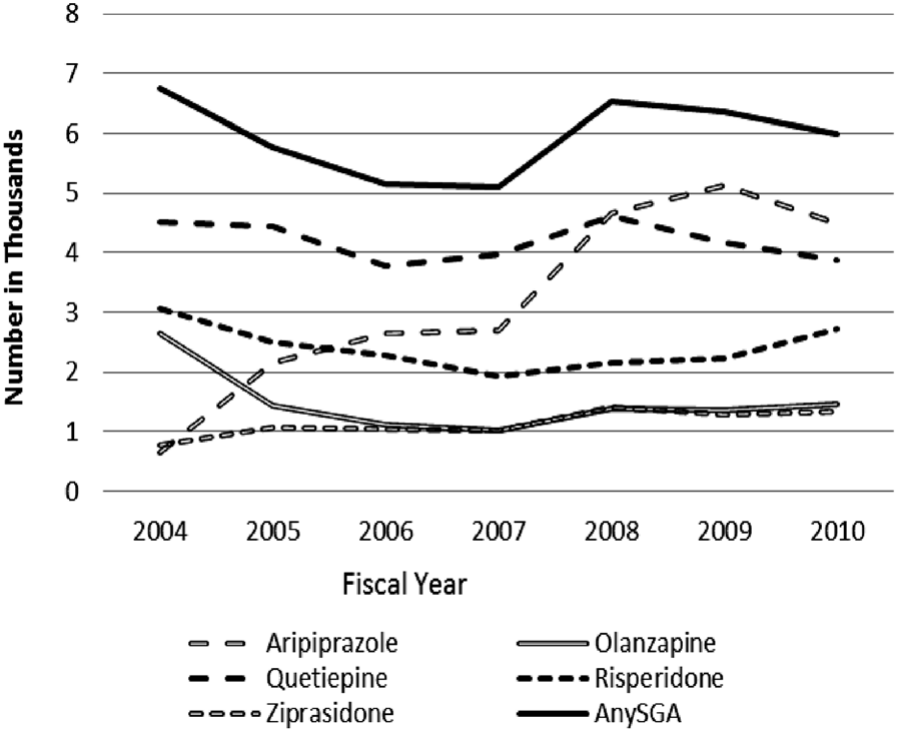
**Patients with bipolar disorder initiating SGAs, 2004–2010.** Note: Figure 2 begins with 2004 since patients were not eligible to be labeled as SGA initiators until month 7 of 2003; see Methods for details.

Figure 2 describes the nationwide number of patients *newly initiated* on an SGA each year, indicating between 5,109 and 6,745 patients entering the population of SGA users each year between 2004 and 2010 (mean = 5,951; 95% CI = 4,685-7,217; *p* < .0001). There was no significant change over time in the number of new initiators per year (annual average increase 11; 95% CI = −332-355; *p* = .9374). When combined with the steady increase in the population of patients with bipolar disorder, this meant that the odds of being newly initiated on an SGA actually decreased as the study progressed (annual OR 0.929; 95% CI = 0.924-0.933; *p* < .0001).

### Description of the patient sample and bivariate comparisons

Table 1 summarizes the VA bipolar population (n = 170,713) including individuals who ever (n = 45,389) or never (n = 81,167) initiated an SGA during the study period, yielding a primary analytic sample of 126,556. The remaining 44,157 patients entered the study taking an SGA and so were excluded from initiator analyses.

**Table 1:** **Description of the sample**

	**Bipolar population (N =170,713)** ^**a**^		**Never initiated SGA during study period (N =81,167, 64.1% of those eligible for SGA initiation)**		**Ever initiated SGA during study period (N =45,389, 35.9% of those eligible for SGA initiation)**	
**Variable**	**Mean**	**SD**	**Mean**	**SD**	**Mean**	**SD**
**Age at study entry** ^**b**^	50.3	13.3	51.6	13.6	48.3	13.0
	**N**	**%**	**N**	**%**	**N**	**%**
**Gender (female)**	24,076	14.1	10,804	13.3	6,954	15.3
**Disability status ≥50%** ^**c,d**^	52,584	30.9	23,067	28.5	13,148	29.0
**Marital status** ^**d**^	76,315	44.9	34,786	43.3	18,265	40.5
**Race/ethnicity** ^**e**^						
White	127,742	82.2	60,381	83.0	34,425	82.6
African American	20,884	13.4	9,305	12.8	5,588	13.4
Hispanic	4,034	2.6	1,861	2.6	891	2.1
Other race/ethnicity^c^	2,684	1.7	700	1.0	455	1.1
**Clinical/treatment course** ^**d**^						
Psychotic features	22,119	13.0	6,822	8.4	7,322	16.1
Bipolar type I (versus type II/NOS)^f^	145,128	85.0	68,344	84.2	40,283	88.8
Antidepressant prescription	139,807	81.9	55,920	68.9	34,969	77.0
Antimanic prescription	128,596	75.3	51,820	63.8	35,755	78.8
Any psychiatric hospitalization	64,117	37.6	23,284	28.7	21,330	47.0
**Comorbidities** ^**d**^						
Diabetes	42,517	24.9	20,679	25.5	10,138	22.3
Obesity	61,182	35.8	27,469	33.8	16,581	36.5
Hyperlipidemia	101,886	59.7	47,770	58.9	26,190	57.7
Substance abuse	79,658	46.7	33,202	40.9	23,280	51.3
Tobacco use disorder	86,311	50.6	37,406	46.1	24,182	53.3
Anxiety disorder	102,130	59.8	43,310	53.4	27,331	60.2
Sleep disorder	29,355	17.2	11,762	14.5	8,072	17.8
Cardiac dysrhythmia	19,600	11.5	9,509	11.7	4,827	10.6
Liver disorder	22,332	13.1	9,460	11.7	5,997	13.2
Kidney disorder	19,044	11.2	9,271	11.4	4,729	10.4
Thyroid disorder^c^	20,226	11.9	9,429	11.6	5,566	12.3
Traumatic brain injury	9,209	5.4	3,629	4.5	2,557	5.6

^a^Includes data on those patients ineligible to be labeled as SGA initiator or SGA non-initiator (N = 44,157).

^b^Unless otherwise stated, all comparisons between the ever-initiated and never-initiated groups were *p* < .0001.

^c^For these variables, comparison between the ever-initiated and never-initiated groups was *p* > .0001 (>50% disability status: *p* = .0385; other race/ethnicity: *p* = .0120; thyroid disorder, *p* = .0006).

^d^Unless otherwise stated, these variables were coded as present if they occurred at any point during the study period.

^e^Based on smaller N, due to missing values for 9% of the sample.

^f^At patient’s last available data during the study period.

Unadjusted bivariate comparisons between SGA initiators and non-initiators using all available data during the study period (Table 1) demonstrated that nearly all characteristics had small but statistically significant associations at the *p* < .0001 level. Patients initiating an SGA during the study period were on average several years younger than non-initiators. Those initiated on an SGA were nearly twice as likely to be identified as having psychotic features, but only slightly more likely to have a bipolar type I diagnosis, substance use disorder, or anxiety disorder, with small, inconsistent impact of medical comorbidities. Patients initiated on an SGA were more likely to have been hospitalized for a psychiatric issue at least once and to receive a non-SGA antimanic prescription at least once.

### Month-by-month, multivariate correlates of SGA initiation

Table 2 presents GLM findings for which, in contrast to Table 1, covariates were entered simultaneously into the model, and were coded as present only if diagnosed simultaneously or within the year prior to the month of SGA initiation. Again, small but statistically significant differences were demonstrated for most variables. Among stronger effects, female gender was associated with reduced likelihood of initiating an SGA (OR = 0.846, 95% CI = 0.815-0.878), as was disability status (OR = 0.740, 95% CI = 0.717-0.763). Compared to the Northeast, patients seen in the South were more likely to initiate an SGA (OR = 1.288, 95% CI = 1.245-1.332), and those in the West less likely (OR = 0.919, 95% CI = 0.886-0.953). Patients with psychotic features were more likely to be initiated on an SGA (OR = 1.696, 95% CI = 1.520-1.891). Patients with a mood stabilizer prescription in the past year were much less likely to initiate an SGA than those who had not (OR = 0.620, 95% CI = 0.606-0.634), unlike the all-years analysis (Table 1). Patients with a sleep disorder diagnosis were more likely to initiate an SGA (OR = 1.555, 95% CI = 1.435-1.685). Most medical comorbidities were only modestly associated with overall SGA initiation rates in our sample. GLM indicated an average reduction in the odds of SGA initiation over time (OR = 0.988 per year, 95% CI = 0.982-0.993. When we added prior hospitalization to the model, it was also strongly associated with SGA initiation (OR = 2.629, 95% CI = 2.546-2.713).

**Table 2:** **Results from GLM predicting SGA initiation**

**Variable**	**Odds ratio**	**95% CI – lower**	**95% CI – upper**	***p***
**Age (decade)**	0.997	0.997	0.997	<.0001
**Gender (female)**	0.846	0.815	0.878	<.0001
**≥ 50% disability status**	0.740	0.717	0.763	<.0001
**Marital status (married)**	1.011	0.985	1.039	.4062
**Race/ethnicity** ^**a**^				
African American	1.068	1.024	1.114	.0021
Hispanic	0.989	0.901	1.087	.8222
Other race/ethnicity	1.096	0.964	1.246	.1614
**Region** ^**b**^				
Midwest	0.991	0.955	1.028	.6221
South	1.288	1.245	1.332	<.0001
West	0.919	0.886	0.953	<.0001
**Clinical/treatment factors in prior year**				
Psychotic features	1.696	1.520	1.891	<.0001
Bipolar type I (versus type II/NOS)^c^	0.968	0.935	1.003	.0736
Antidepressant prescription	0.903	0.882	0.924	<.0001
Antimanic prescription	0.620	0.606	0.634	<.0001
**Comorbidities in prior year**				
Diabetes	0.919	0.882	0.957	<.0001
Obesity	0.958	0.913	1.004	.0739
Hyperlipidemia	0.991	0.960	1.023	.5860
Substance abuse	0.965	0.933	0.999	.0407
Tobacco use disorder	1.065	1.030	1.103	.0003
Anxiety disorder	1.082	1.051	1.114	<.0001
Sleep disorder	1.555	1.435	1.685	<.0001
Cardiac dysrhythmia	1.096	0.999	1.202	.0520
Liver disorder	1.145	1.079	1.214	<.0001
Kidney disorder	1.187	1.069	1.318	.0014
Thyroid disorder	1.021	0.956	1.089	.5372
Traumatic brain injury	1.109	0.944	1.303	.2068
**Change per year**	0.988	0.982	0.993	<.0001

^a^Comparison: White.

^b^Comparison: Northeast.

^c^Patient was labeled as bipolar type I for a given month if at least 10% of bipolar encounters to date were for bipolar type I. See text for details.

### The role of clinical complexity in SGA initiation rates over time

We hypothesized *a priori* that SGAs were being initiated for an increasingly diverse population over time. Variable-by-time interaction terms for several indicators of severity (selected *a priori*) were added to the GLM. These analyses indicated that, over successive years, SGA initiation became increasingly common for individuals with: type II/NOS (beta = 0.0168, Z = 2.34, *p* = .0219), no antimanic prescription in the past year (beta = 0.0396, Z = 7.98, *p* < .0001), and no previous psychiatric hospitalization (beta = 0.07, Z = 12.78, *p* < .0001), although not for those without psychotic features (beta = 0.0168, Z = 0.83, *p* = .4196). Taken together, these results indicate that, over time, patients with less severe bipolar disorder (as marked by an absence of previous hospitalizations, psychotic features, antimanic prescriptions, and bipolar type I diagnoses) represented an increasing share of new SGA initiations.

### Association of comorbidities with specific SGAs

Consistent with *a priori* hypotheses, diabetes, obesity, and hyperlipidemia were associated with decreased odds of initiating olanzapine, quetiapine, or risperidone compared to aripiprazole (ORs ranging from 0.352 to 0.856, all *p* < .0003) (Table 3). Cardiac dysrhythmia was associated with a trend toward lower likelihood of initiating ziprasidone (OR = 0.725, 95% CI = 0.505-1.042, *p* = .0825). Individuals with substance use disorders were more likely to initiate olanzapine (OR = 1.321, 95% CI = 1.230-1.417, *p* < .0001) or quetiapine (OR = 1.363, 95% CI = 1.288-1.442, *p* < .0001). Those with comorbid sleep disorder did not significantly differ in use of any SGAs with the exception of risperidone (OR = 0.794, 95% CI = 0.654-0.965, *p* = .0204).

**Table 3:** **Multinomial regressions for initiation of individual SGAs, compared to aripiprazole**

	**Olanzapine**				**Quetiapine**				**Risperidone**				**Ziprasidone**			
**Comorbidity in the past year**	**Odds ratio**	**CI – lower**	**CI – upper**	***p*** **-value**	**Odds ratio**	**CI – lower**	**CI – upper**	***p*** **-value**	**Odds ratio**	**CI – lower**	**CI – upper**	***p*** **-value**	**Odds ratio**	**CI – lower**	**CI – upper**	***p*** **-value**
Diabetes	0.477	0.428	0.531	<.0001	0.771	0.718	0.828	<.0001	0.856	0.789	0.929	.0002	1.05	0.939	1.174	.3945
Obesity	0.352	0.305	0.407	<.0001	0.657	0.602	0.716	<.0001	0.607	0.547	0.675	<.0001	1.109	0.974	1.262	.1181
Hyperlipidemia	0.686	0.632	0.744	<.0001	0.857	0.808	0.91	<.0001	0.838	0.781	0.898	<.0001	0.944	0.855	1.041	.2469
Substance abuse	1.321	1.23	1.417	<.0001	1.363	1.288	1.442	<.0001	1.216	1.14	1.298	<.0001	0.987	0.896	1.088	.7917
Sleep disorder	0.860	0.695	1.065	.1666	1.158	0.991	1.353	.0655	0.794	0.654	0.965	.0204	1.05	0.809	1.362	.7140
Cardiac dysrhythmia	1.422	1.137	1.778	.0020	0.971	0.800	1.179	.7690	1.116	0.9	1.385	.3166	0.725	0.505	1.042	.0825

## Discussion

This study revealed several core findings. First, SGA use for bipolar disorder is increasing within VA medical centers, with an average of about 6,000 new SGA initiations each year. Time trends suggest that this growth rate was remarkably steady from 2004 to 2010, with no year seeing fewer than 5,000 or more than 7,000 new initiations. Given a roughly parallel increase in the overall bipolar population treated within VA, the proportion of individuals with bipolar disorder receiving an SGA prescription in a given year rose from 59% in 2003 to 75% in 2008 with a slight decrease to 72% in 2010. These increases in use may have in part been driven by recent treatment guidelines that suggest SGAs as potential first-line treatments for multiple phases of bipolar disorder [15,16]. This decrease in overall use after 2008 may in part reflect effects of toxicity warnings on prescribing practices from the middle 2000’s [43]. Regarding specific SGAs, use of aripiprazole, quetiapine, and ziprasidone increased over time while use of olanzapine and risperidone declined. Among providers with prescription privileges treating patients with bipolar disorder, the proportion using SGAs shrunk somewhat over the course of the study (from 90% to 86%), though still encompasses the vast majority of potential prescribers.

A novel innovation in these analyses is the use of GLM techniques to investigate factors that affect provider decision-making on a month-by-month basis, to focus on clinical data available to prescribing clinicians. These analyses revealed that, while the number of new initiators each year remained relatively steady, the odds of initiating an SGA actually decreased slightly over the course of the study (OR = .988 annually). Additionally, two related factors emerged as key in predicting initiation of SGAs. First, SGA initiation was more common overall for patients who appeared more clinically complex. Second, over time SGA use appeared to be spreading to those with less complex clinical profiles.

### SGA initiation more common for clinically complex patients

Providers were more likely to initiate SGAs for patients who were generally more psychiatrically ill at the time of their prescription (i.e. more likely to have a history of psychiatric hospitalization, recent psychotic features, or recent sleep disorder diagnosis); this is consistent with our finding that those who received an SGA also—at some time during the study period—received another antimanic (Table 1). However, on a month-by-month basis, prescriptions of other antimanics were less likely prior to initiation of SGAs. One interpretation is that those not receiving SGAs were already effectively controlled by a non-SGA medication. However, it is also possible that SGAs are being increasingly utilized as first-line treatments, bypassing other medications. Definitive answer awaits finer-grained treatment trajectory analyses.

### SGA initiation spreading to less clinically complex cases

Time trend analyses indicate that, even though SGA initiation was more common for more complex cases as above, there was a broadening over time of the use of SGAs to include less clinically complex populations. Specifically, variable-by-time interaction terms indicated that SGA initiation became increasingly common for those with bipolar type II/NOS, those with no concurrent antimanic prescription, and those with no prior psychiatric hospitalization. This suggests that over time VA providers may have become increasingly comfortable prescribing SGAs for less severe portions of the bipolar population, though more provider-specific quantitative or qualitative data will be needed to draw firm conclusions.

### Other factors related to SGA initiation

Most patient demographic covariates showed only modest associations with SGA initiation. African American patients were somewhat more likely to be given an initial SGA prescription during the study period compared to Whites, which is broadly in line with another VA study which found slightly but nonsignificantly higher rates of SGA use among African Americans (52%) compared to others (44%) with bipolar disorder [34]. Given evidence from previous studies that racial differences in SGA prescriptions may be closing [35], future studies should consider including race by time interaction terms in their statistical analyses.

The effects of medical comorbidities on SGA initiation appeared relatively small when results for all SGAs were combined, which makes intuitive sense given the heterogeneity of cardiometabolic risk profiles among the SGAs we studied. As expected, certain medical comorbidities were associated with substantial differences in prescribing practices for specific SGAs. Notably, individuals with preexisting diabetes, obesity, or hyperlipidemia were less likely to receive those SGAs with more prominent cardiometabolic effects (olanzapine, quetiapine and risperidone). Patients with substance abuse, in contrast, were more likely to receive these drugs: the sedating effects of these SGAs may be seen as desirable in treating patients with substance abuse disorders in whom benzodiazepines are considered inadvisable [39]. Contrary to expectations, quetiapine was not statistically more likely to be prescribed than aripiprazole for individuals with sleep disorders. It should be noted, however, that providers would only be likely to provide a sleep disorder diagnosis if a patient’s sleep troubles occurred independently of their bipolar condition (e.g. sleep apnea), which may reflect a complex population in whom sedating drugs would be avoided.

Regional variation in SGA initiation after controlling for covariates was marked by higher rates of SGA use in the South. Veterans in this region of the country have lower self-reported mental and physical quality of life than other regions, perhaps reflecting poorer clinical course or sociodemographic differences [40,44]. However, analyses both in this bipolar population and among veterans with PTSD [39] indicate prominent regional variation despite extensive control for demographic and clinical characteristics. This finding may thus reflect regional differences in provider practice patterns, commonly reported across many health domains [45].

### Limitations

Although administrative data provided a very large sample (n > 125,000 patients), it has the disadvantage of relying upon clinicians’ diagnostic coding accuracy. Some conditions (e.g. obesity, sleep disturbance) may have been present and influenced treatment decisions and yet not been coded by clinicians. Similarly, codes for bipolar mixed episodes were not reliably utilized, although patients suffering mixed manic and depressive symptoms may be more responsive to SGAs than to other antimanic medications [46–48]. Furthermore, we could not include data on non-VA prescribing. In addition, we opted to approach these analyses primarily as class analyses, although we secondarily conducted *a priori* analyses on specific SGAs and specific clinical correlates. Our current analyses did not include data on provider variables (e.g. demographics, academic affiliation, training background), but this is a clear direction for future research. Finally, our primary comparison was between patients initiating SGAs and those not initiating SGAs; we did not conduct additional analyses with different comparison groups (e.g. patients initiating lithium or other antimanics). Future research on SGA initiation exploring provider-level variables and using these other comparison groups would help shed further light on prescribers’ initial medication decision for bipolar disorder.

## Conclusions

Increase in SGA initiation within VA appears to be driven by an increase in the bipolar population rather than increasing rates of use of these drugs. Our month-by-month analyses, however, indicated that initiation rates are related to three factors: clinical complexity, spread of SGAs to less severe patients over time, and geographic variation that cannot be accounted for by our extensive list of covariates. Assessing the clinical appropriateness of SGA use is beyond the scope of these analyses, and must be judged in the context of the evolving clinical evidence base as applied to individual patients at the time of specific treatment decisions. However, the regional heterogeneity in particular suggests that social [49] or administrative [50] factors may play a role in the spread of SGAs for bipolar disorder. Thus, attention at the clinical, administrative, and policy levels may be warranted to ensure that utilization of SGAs is driven primarily by evidence and patient need rather than other factors.

## Abbreviations

SGA: Second-generation antipsychotic
VA: Department of Veterans Affairs
